# Memoir on a Periodical Difficulty of Breathing, Tending to Prove the Influence of the Moon on the Human Body

**Published:** 1802-11-01

**Authors:** Don Antonio Tranzieri

**Affiliations:** Physician to the Royal Family, and President of the Royal Academy, of Madrid


### *Memoir on a periodical Difficulty of Breathing, tending to prove the Influence of the Moon on the human Body; by* DON ANTONIO TRANZIERI, *Physician to the Royal Family, and President of the Royal Academy, of Madrid.*

FIRST PERIOD. IN the year 1775, a lady,* of bilious temperament, *dry* conſtitution, and great nervous *ſusceptibility*, with abundant flow of the menſes, who had enjoyed throughout her life but a feeble ſtate of health, experienced at her fortieth year, in the month of September, a difficulty of breathing ſimilar to aſthma, but which did not interfere with her ordinary habits. This attack continued two days; a ſhort time after the ſame recurred, and continued as before, two days. The only apparent cauſe which had preceded this affection was a great fright. After three ſimilar attacks, a fourth was accompanied with ſuch an oppreſſion and ſenſe of tightneſs about the cheſt, that the patient prayed ſhe might be opened, and with her hands made efforts as if to accompliſh it herſelf. In this ſtate, not a drop of fluid could be got down; and if an attempt was made to moiſten her throat, dried by the frequency of reſpiration, ſhe ſeemed as if ſuffocated; this was accompanied by profuſe ſweats from the forehead and breaſt, a violent pain in the back, and the moſt violent hoarſe ſhrieks; her reſpiration ſometimes became ſo ſuddenly quick as to render it impoſſible for her to ſupport her conſciouſneſs for any time; this was immediately followed by fainting; her breathing as well as her ſenſes, internal and external, became completely ſuſpended, ſo as that ſhe might be taken for dead, was it not for her pulſe, which never altered from the natural ſtate. On water being thrown in her face, in order to recover her from this apparent death, ſhe came immediately to herſelf, but the ſame train of ſymptoms were ſure to follow, quick breathing, fainting, and ſuſpenſion of ſenſibility. Theſe changes occupied nearly two hours, after which the patient ſeemed in the ordinary ſtate of a perſon with aſthma; ſhe could throw herſelf on the bed, and reſt for ſome hours only, for the ſame ſymptoms were renewed frequently in the ſpace of two days; after which they all diſappeared, the patient found herſelf perfectly well, and her reſpiration as free as in the uſual ſtate of health. This continued for ten or twelve

---

* Donna Maria-Franciſca de Parte-Arrozo y Avendanno, widow of Signior Don Franceſco Eduardo Paneagua, of His Majeſty's Council, and Secretary for the Affairs of India.

days, when, without any apparent caufe, the fame attack, the fame fymptoms, and the fame intermiffions that have been already defcribed, occurred.

THE RETURN. The return of the paroxyfms after a certain number of days, without any apparent caufe, led me to conjecture that this regularity of effect might be owing to lunar influence; I thought it worthy of attention, though I gave at that time no great credit to the idea; I confulted the almanack in order to afcertain the day of the moon, which correfponded to the exifting paroxyfm, and I found it to be the day before the eve of the full moon. I reflected a little, and recollected that the previous paroxyfms occurred exactly within the two days preceding the new and full moon; neverthelefs, I paffed over the two days of the exifting attack, and waited for further proof, for the day before the eve of the new moon. The time arrived, I perceived that the paroxyfm took place exactly on that day; fince when, the conftancy of recurrence has fully convinced me, that the renewal of thefe fymptoms was in fact owing to the influence of the moon. The days of intermiffion, counted from the very day of the new moon to that preceding the eve of the full one; and from the day of the full moon to the day before the eve of the new one: On the day preceding that which I have marked for the attack, the patient experienced a certain degree of oppreffion throughout the cheft; it was the certain fign of the approaching dyfpnœa which was to occur on the decline of the following day; and it was then that the patient was obliged to take to her bed. The accefs of orthopnœa occurred between nine and eleven o'clock at night precifely; during the remainder of the night and following day, the difficulty of breathing continued, but fo as to enable her to remain in bed, and free of every other kind of pain, till nine o'clock at night arrived, which brought on the orthopnœa as in the night before, and for the fame period of two hours. The following day, at day-break, that is, on the day of the new or full moon, her refpiration became natural, fhe quitted her bed, and continued well till the fame period of the next change. Such violent and formidable attacks could not fail to create confiderable changes in the animal economy. The days of intermiffion were changed into days of pain and fatigue; to the fymptoms of orthopnœa, others became affociated, fo that during the intermiffion the weaknefs became fo great, and the organs of refpiration fo fufceptible of compreffion, that on making the leaft effort to move or get out of bed, the breathing became fo quick, that if the patient did not remain quiet, fhe would rifk fuffocation; fhe could not even fuffer the trifling motion of fewing or knitting, without her refpiration being immediately affected.

In

In the depth of winter, and in the heat of ſummer, the patient paſſed few days out of bed, on account of the conſtant difficulty of breathing which ſhe experienced. In the temperate ſeaſons only ſhe paſſed the days of intermiſſion with leſs fatigue. The legs, thighs, and belly ſwelled; the urine came in ſmall quantity; her diſreliſh for food amounted to diſguſt; her thirſt was exceſſive, and not to be moderated by any drink, and ſo teaſed the patient that ſhe preferred the torments of the paroxyſm which promiſed to cloſe her ſufferings. She refrained, it is true, from drink, not in the apprehenſion of increaſing the ſwelling, but becauſe her thirſt was no way relieved by drinking; the throat and lips were dry, the tongue was not ſo, but the patient always complained of a ſenſation as if the end was charged with pepper; to theſe was added a very abundant fluor albus, extremely acrid and excoriating. In the orthopnic ſtage of the paroxyſms the foregoing ſymptoms alternated with a kind of drowſineſs or fainting, accompanied by difficult and ſtertorous breathing. This continued five or ſix minutes, and went off with a yawning, after which came on the quick reſpiration, fainting, and other ſymptoms. They were equally provoked on tickling the noſe by any ſmall roller ſteeped in vinegar, a remedy we had of her nurſe when the yawning continued, ſo as to threaten lethargy. In theſe changes the patient paſſed the melancholy period of eleven hours; and it is remarkable, that till the tenth hour, the force and violence of the paroxyſm ſuffered no diminution, and that this hour was waited for with impatience, in order to reſcue the patient from imminent danger, which threatened her life. During more than four years paſſed in theſe torments, no fever had ever manifeſted itſelf; but towards the commencement of January 1780, a quotidian fever came on at night with ſlight tremors; the heat was conſiderable, but there was neither thirſt nor dryneſs of the tongue; the reſpiration, though a little quickened, was not ſo much ſo as in the acceſs of aſthma, and only in proportion to the frequency of the pulſe and the ſtrength of the fever: this diminiſhed after midnight, and diſappeared at day-break, accompanied by a gentle but general perſpiration. This fever continued for ſix months, coming on every day at the approach of night, except on the days of the lunar paroxyſms. It ſometimes happened, however, that the fever appeared on one of the nights of this paroxyſm; it was always the ſecond; and the orthopnœa did not make its appearance on that night from nine to eleven. The menſtrual diſcharges never failed to appear at their uſual time: they generally continued from ſix to eight days; and when they happened to fall on the days of the lunar paroxyſms, the evacuation ſtopped, and did not reappear till the end of the attack, when they again came on, and remained their ufual time; the fluor albus was equally affected, but again followed its courfe on the difappearance of the complaint. I never judged it prudent to make ufe of any remedy to carry off this nightly fever: in fact, perceiving that it did not aggravate the difeafe, I thought it better to do nothing, and I even conceived it capable of lightening the preffure of fo much fuffering. One thing is certain, that after fix months continuance of the fever, the fwelling of the legs, thighs and belly, difappeared; the urine became more abundant, the fluor albus lefs in quantity and lefs acrid, the thirft more moderate, the appetite better, and the vigour of the feveral motions more fenfible; the patient could walk without fatigue; fhe got fat, and a more healthy colour; in fhort, the influence of external caufes on her refpiration, except that of the moon, became fenfibly lefs. No amelioration was obferved in the paroxyfms of orthopnœa, which always continued with the fame intenfity at the lunar periods, except when the fever came on the fecond day of the attack. This, however, was very feldom: the calm of the periods of fuffering did not become greater; the reafon of which we fhall perceive in the details of the fecond ftage.

Second Period. The patient was now in her forty-feventh year, and in the fifth from the commencement of her complaint, at which time fhe perceived an irregularity in her menftrual difcharges, which ufually indicate the total difappearance of thefe evacuations; the interval of a month, fometimes two, occurred; fometimes they came on twice in the fame month, at one time in profufion, at another in fmall quantity. During this irregular menftruation, fhe experienced new and increafed pains; fhe was feized with violent pains about the navel, which extended to the loins and fhoulders, and which continued two or three hours at a time; thefe came on at uncertain periods, and followed no regular courfe; on their appearance the fluor albus, as well as the menftrual difcharge, became fufpended, and thefe evacuations returned as foon as the pains went off. What proved ftill more diftreffing was, that whenever the pains became very violent (a circumftance which often happened) they brought on dyfpnœa and orthopnœa, and difappeared immediately. Whether thefe pains preceded the orthopnœa or not, this laft never failed to occur at its ftated period; but one circumftance remarkable was, that the fleepinefs and fainting which followed or alternated with the agonies of difficult refpiration, difappeared entirely; they were replaced by convulfive motions of the trunk, head, arms, and hands; the contractions of the hands were fo ftrong as to require very confiderable force to open them. In the height of fuffocation, the the patient, as if in defpair, would make efforts to get out of bed, ftrike her head againft the wall, and her head and breaft with her hands clinched; and would ftruggle with the greateft violence to difengage herfelf from thofe who held her. In thefe convulfive motions the difficult and accelerated refpiration was not near fo violent as before. It would feem as if the complaint was divided between her organs of refpiration and the the mufcles of her extremities; and the proof of this was, that when the convulfions did not exift, or were moderate, the accefs of orthopnœa refumed its ufual violence as on the firft period of the complaint. It was remarked too, that during the accefs of orthopnœa, if fhe happened to perceive water near her, or if water was applied to wet her lips, fhe immediately experienced a trembling from the horror fhe conceived. This averfion to water is very remarkable; it continued all the time of the paroxyfms, notwithftanding her thirft; but this paffed, fhe experienced in drinking the moft delicious pleafure.

These were not the only circumftances which the fecond period prefented. A repetition of convulfive motion and of orthopnœa, had given fuch a delicacy and fufceptibility to all the organs of refpiration, that the fight of a rat, a flight difguft, a change in the atmofphere, excited immediately a difficulty of breathing on the days of intermiffion. The ringing of bells fomewhat ftrong, occafioned, gradually, the dyfpnœa, which augmented till it terminated in orthopnœa that threatened her life. This effect did not difappear till long after the bells had ceafed to ring; and as this lady lived in the Place del Cordon, and very near the two towers of the parochial churches of St. Peter and St. Juft, I had frequent opportunities of obferving the effects which the bells produced: thefe often occurred, on account of the numerous feafts celebrated in thefe two churches. The precaution of fhutting the windows and doors, anfwered no purpofe, the noife was always fufficiently ftrong. Notwithftanding every meafure which could be taken to fhorten the duration of thefe peals, no redrefs could be had; they were committed to the care of boys, who never let them reft; it was therefore ufelefs to complain. In this dilemma there came into my mind an expedient to counteract fuch unpleafant effects, which was followed with fuccefs. I conceived it probable that this noife of bells might be counteracted by fome other more agreeable to the patient, fo as to be protected from the influence of the firft. I arranged it fo, that, during the ringing of the bells, a perfon fhould remain near her with a mandoline; and while he played, accompany the inftrument with his voice, without the leaft intermiffion. This agreeable noife rendered her infenfible to the ringing of the

 bells, bells, and prevented completely the difficulty of breathing. On the other hand, the dyfpnœa certainly returned, if by hazard or inattention the ringing began before the mufic; but even then, the found of the mandoline, although rather too late, was ufeful, even neceffary, to ftop the progrefs of the dyfpnœa, which, without it, would become more violent, even dangerous, in proportion to the continuance of the ringing. It would appear incredible that this lady could have refted for five years under the preffure of fuch accumulated fufferings, fo much the more as there was fuperadded a continual palpitation of the heart. It was fortunate that at this period, that is, towards the middle of January 1786, and in the height of her miferies, that the nightly fever made its appearance. The happy effects which followed it in the preceding period, made it be confidered a good omen, and as the dawn of her tranquillity. It came on about the decline of the day, with the fame fymptoms as before, and went off at day break of the following day. Its periodical return continued four months, and difappeared towards the end of May. It is neceffary to mention here, that the fever did not take place the two days of the lunar paroxyfms; it was obferved, however, that it fhowed itfelf many times during the fecond night, and even once or twice the firft, and that then it had the effect of fufpending the attack of orthopnœa, which ufed to occur between nine and eleven o'clock. In the firft period the fever was never obferved the firft night. In proportion as the accefs of fever came on, the patient experienced a relaxation in her fufferings, fo much fo, that the pains in her loins and belly difappeared; the impreffion which the found of bells produced was much diminifhed, as well as thofe of external caufes that ufually brought on dyfpnœa and orthopnœa in the days of intermiffion; the convulfive movements which attended the accefs of orthopnœa, had no longer either the fame degree of violence or of danger; the palpitations of the heart became extremely moderate; and laftly, her menftrual difcharges, as well as her fluor albus, completely difappeared.

THIRD PERIOD. After fo great and happy a change operated by the fever, it fhould feem that this difpofition of the body, which made it fo fufceptible of lunar influence, was to ceafe, and be replaced by a ftate that had no relation to this influence. This effect was not altogether obtained; neverthelefs, it was found that the intenfity of the paroxyfms of the afthma was growing gradually lefs; they were changed too in the order of their coming on, as well as in their duration. From the beginning of this period the lunar paroxyfms, which conftantly conftantly manifefted itfelf at the decline of day, and the day before the eve of the full and new moon, declared itfelf at day-break of the third day, before the lunar changes. In the fpace of a year, it anticipated them another day, fo that the duration of the lunar paroxyfm was then four days, beginning at day-break of the fourth day before the change, and terminating at day-break of the full or new moon; having, however, this peculiar circumftance, that during more than ten years, it never happened that the orthopnœa came on within the two firft of thefe four days, but always in the nights of the two days preceding the lunar changes. In the fame manner, from the commencement of this period, the orthopnœa had this alteration, it came on an hour earlier; fo that inftead of nine to eleven, it was from eight to eleven at night.

Let us continue the hiftory of the difeafe, fuch as it appeared throughout this period. At day-break of the fourth day, before the lunar change, the refpiration became fomewhat fhorter and more frequent: this obliged the patient to remain in bed, for when fhe tried to get up fhe became much fatigued. This difficulty neither increafed nor diminifhed until within a few minutes before eight o'clock of the day before the eve of the change. The patient then felt all over the body a certain anxiety, extremely teazing, and like to what is called *inquietudes*. She felt a fentiment of oppreffion about the cheft with frequent yawnings, which tended to quicken refpiration, and went on till they at laft terminated in the ftate of orthopnœa; that is, fhe could not breathe but when feated. A little after eight o'clock her refpiration was fo quickened, that in the fpace of five feconds it repeated ten or twelve times or more; this lafted for half a minute, or rather more: had it continued a little longer, fuffocation muft have been the confequence. This very rapid refpiration became fufpended for five, fix, or eight minutes, and was renewed in the fame manner. Thefe alternations lafted two hours, never lefs than one hour and a half. While in this ftate, the head moved with the fame celerity, fometimes upwards, fometimes downwards;* the mufcles of the neck attached

* To moderate this violent motion of the head, it was faftened with a band that went from the forehead to the occiput; a precaution by no means ufelefs. But this advantage was of fhort duration, for the conftant elevations and depreffions of her head did not permit the bandage to confine her equally; we were obliged to fix the head by placing a hand on the forehead and another behind: In this way the head of the patient was compreffed whenever attached to the occiput, were ſo hard and ſtiff that they reſembled a hard cord, which ſwelled and contracted with great rapidity. Contractions in the muſcles of the lips and noſe were equally perceivable, but not with the alternation of relaxation, as in thoſe of the neck: No doubt, the ſame contractions exiſted in the other muſcles of the face, as there flowed tears with ſneezing and running from the noſe. The patient felt, all over the forehead and head, a ſenſe of tightneſs or conſtriction;—the mouth and throat were dry. In this ſtate ſhe could not utter a word, ſwallow her ſpittle, or let down a drop of water: She often ſighed in a plaintive and lamentable way, ſufficiently loud to be heard at a conſiderable diſtance. In this ſtate her pulſe never varied from their natural ſtate; the intermiſſion of ſome muſcles, of which we have already ſpoken, was more or leſs ſhort, according as the yawning or cough came on, or as the patient tried to turn in her bed, to ſwallow her ſaliva, &c. The moment that any of theſe motions took place, her reſpiration became quick: So certain was it that yawning was the moſt frequent cauſe of this repetition, that in this caſe all the accidents were multiplied; and whenever a yawning occurred, without being followed by theſe accidents, it was the moſt infallible ſign of the ceſſation of the ſymptoms for that night. This happened after the term of two hours, at the expiration of which the reſpiration became quiet as before; the patient could turn herſelf to whatever occupation ſhe pleaſed, to eat, drink, &c. This calm continued till eight o'clock the following night, when the ſymptoms already deſcribed returned; but on the approach of the day of the new and full moon, the patient became well and her reſpiration natural. After continuing a year in this ſtate, it was remarked, that the acceſs of orthopnœa of the ſecond night; that is, the one on the eve of the lunar change, did not come on, provided there was, at this period, no eclipſe of the ſun or moon, in which caſe the attack never failed. There occurred alſo new nocturnal fever in the winter ſolſtice of 1787, which continued three months.

It was rare that the fever failed in the night of the day before the eve of the change, then only the acceſs of orthopnœa manifeſted itſelf from eight to ten o'clock; at length, after repeated fevers, this acceſs of fever on the firſt day failed a conſiderable number of times; however, when there was an eclipſe of whenever ſhe required it, and to a certain degree made the motions of her head and of reſpiration to ceaſe, to the great aſtoniſhment of the aſſiſtants.

of the ſun or moon, the acceſs conſtantly took place, but then there was none the ſecond night.

Towards the end of the year, 1788, the acceſs of orthopnœa ceaſed entirely; it was only under the following circumſtances that it returned: Firſt, when the patient was ſeized with great diſguſt, or a ſenſe of weight in the beginning of the paroxyſm. Secondly, when in the beginning, or in the courſe of the paroxyſm, ſhe experienced great melancholy without any evident cauſe. Thirdly, when in the courſe of the paroxyſm ſhe felt great averſion to water. Fourthly, and principally, when there happened an eclipſe of the ſun or moon. Soon after, the acceſs of orthopnœa failed once or twice, notwithſtanding theſe eclipſes of the ſun, and at length it failed entirely on theſe occaſions. The patient had not this good fortune in the eclipſes of the moon, till 1793; and until this year theſe eclipſes warranted us to predict with certainty the acceſs on the night before the eve of the change of the moon. Towards the winter ſolſtice of this year ſhe was attacked with another fever, which continued the entire month of January following. The 14th of February, the day on which the firſt eclipſe of the moon occurred for the year 1794, the orthopnœa failed for the firſt time; the ſame thing happened on the ſecond and laſt eclipſe of the year on the 21ſt of Auguſt; ſo that, in this year, there was no acceſs of orthopnœa. There occurred one however during the eclipſe on the 3d of February, 1795, but leſs violent than uſual; from that moment to this (the end of October, 1796) the paroxyſms of aſthma have followed regularly in the new and full moon, with the ſame punctuality in the days and hours of attack, and diſappearing without being accompanied by any acceſs of orthopnœa, either in the eclipſes of the ſun or moon. However, in the two nights which precede the new and full moon, there always appears, between the hours of eight and ten, a ſlight degree of frequency in the reſpiration, with ſome yawning and uneaſineſs in the body, eſpecially when there is an eclipſe. In other reſpects this lady is become active and lively, her complexion has become healthy, and ſhe has acquired as much fleſh as can be expected in a perſon naturally of a thin habit. She no longer experiences any diſagreeable impreſſion from exceſs of heat or cold, from the changes in the weather, or other circumſtances, which formerly ſo eaſily renewed her difficulty of breathing, out of the ordinary periods of attack. On the whole, at the age of ſixty-four years, ſhe enjoys a health and appearance that one has little reaſon to expect in a perſon, after ſuch long and painful ſufferings. I ſhould not here paſs over in ſilence a very ſingular phenomenon, which has been remarked five years back, and which ſtill exiſts, namely, that on the day which which precedes the paroxyſm, there appears on the edge of the noſtril, a ſmall puſtule, the inflammation and ſuppuration of which continue during the four days of the paroxyſm, after which it dries.

To conclude the hiſtory of this complaint, I ſhall relate the effects of ſome remedies which were employed. In the paroxyſm, bleeding, antiſpaſmodics, and all remedies, as well external as internal, were without any effect. The difficulty of breathing never went off before the uſual term of the paroxyſm. But out of the paroxyſm, that is, in the interval of the two, the anodynes, antihyſterics, &c. produced no effect on the acceſs which might come on during this interval. They nevertheleſs gave way to bleedings; ſo much ſo, that however difficult the breathing, the patient had not loſt two ounces of blood, before ſhe reſpired deeply and freely as in the natural ſtate, and cried "At length I am eaſy." If the difficulty of breathing failed once or twice to give way at the moment of letting blood, in a quarter or half an hour at moſt, the patient found herſelf in her natural ſtate; and experience had taught, that to bring about this calm, two ounces of blood were enough; this quantity therefore was never exceeded. In one of the intervals of the firſt period, there came on a violent attack of orthopnœa, which returned every day, beginning at three o'clock in the day and finiſhing after four. In the intermediate hours bark was given; half an hour after the firſt doſe, the attack took place, and went off entirely at the end of two hours. The ſecond doſe had the ſame effect. It was conceived that the action of the bark on the ſtomach provoked the difficulty of breathing; it was propoſed to be given in clyſters, in doſes of half an ounce, and repeated every three hours; by this means the acceſs was ſhortened. Encouraged by this obſervation, it was ſuggeſted, that if the patient took the bark during the time from one paroxyſm to another, in ſtronger doſes, and more frequently repeated on the days which preceded the occaſion of the lunar paroxyſm, that it might be totally prevented. This was done, but without effect, the paroxyſm came on at the uſual hour, with the ſame force and circumſtances as before; and it was not obſerved either at this or any other time that the patient took bark by the mouth, that the reſpiration was in the leaſt affected by its preſence in the ſtomach. With reſpect to potential cauteries, we were always obliged to renounce them, the ſenſibility of the ſkin was ſuch that the patient could not even ſuffer a ſlight friction on the legs or back without immediately experiencing a difficulty of breathing, or an increaſe of it, if this ſtate already exiſted; the reſpiration even being in its natural ſtate, the ſimple action of of rubbing foftly the legs, or wafhing them with warm water, was fufficient to hurry the refpiration, fo that it was neceffary immediately to defift, in order to permit the patient to repofe herfelf and take breath.

To thefe details which compofe the hiftorical part of the Memoir publifhed by Dr. Tranzieri, we muft add thofe which relate to the termination of this fingular complaint, and which he communicated to Cit. Hallé, and have been inferted in the Magazin Encyclopedique.

"When I publifhed the hiftory of the fingular difeafe of Madame de Parte-Arrozo, (fays Dr. Tranzieri) we were near the end of October, 1796. The accefs of afthma correfponding to the lunar changes, continued to recur without the leaft interruption, and to be renewed at the periods correfponding to the full and new moon, until the new moon of the 17th of March, 1798; in the full moon immediately following, of the 21ft of the fame month it failed, and has not fince taken place, now eighteen months. It is neceffary to obferve, that eight months before the difappearance of the attack, the patient experienced confiderably violent pains in the left fide of the head; the eye of that fide wept, and a quantity of water flowed; the fight became altered and obfcured, and at this time a cataract was perceived. When the cataract was complete, the vifion became completely intercepted on that fide, and at this time ceafed entirely; the periodical returns of the afthma correfponding to the lunar changes ceafed. In proportion as the cataract became more complete, the difficulty of breathing in the time of the paroxyfm became lefs great, and before this it never was obferved fo much as diminifhed. Throughout this infirmity, (adds Dr. Tranzieri) if we except the firft year, we made ufe of no remedy which could derange the courfe of Nature. If they had been reforted to; above all, if we had ufed all the refources of the Materia Medica, and none of which feems calculated to deftroy the caufe, abfolutely unknown, of this difeafe, fhould we have been able to obferve fo well the refources of Nature? Would the patient have furvived? This cure is therefore a benefit conferred by Nature, and by Nature only does this lady, at the age of fixty-feven years, (and fhe does not appear to be fo old) enjoy without the leaft inconvenience, a health which promifes her yet a long life."

